# Effect of Pharmacist Home Visits on Weight Control in Overweight Elderly Hypertensive African American Patients: Managing Your Blood Pressure (My Bp) Program

**DOI:** 10.9734/jamps/2021/v23i730249

**Published:** 2021-10-25

**Authors:** Ivy O. Poon, Kimberly Pounds, Aisha Morris-Moultry, Terrica Jemerson

**Affiliations:** 1 Texas Southern University, 3100 Cleburne Ave., Houston, TX 77004, USA

**Keywords:** Hypertension, overweight, African Americans, home-visits, pharmacist

## Abstract

**Objective:**

The objective was to investigate the effect of a home-based pharmacy intervention on body mass index (BMI) in a cohort of older hypertensive overweight African American (AA) patients.

**Design:**

A secondary analysis of data collected in a community-based intervention study.

**Setting:**

Community-based.

**Participants:**

AA patients, ≥ 65 years old, residing independently, with hypertension diagnosis and BMI ≥ 25.

**Interventions:**

During a 6month period, patients received 1) two in-home pharmacist-led consultations on weight management, 2) bi-weekly telephone counseling, and 3) health education strategies.

**Main Outcome Measures:**

BMIs at baseline and 6 months; stages of behavioral change in diet and exercise based on the Transtheoretical Model.

**Results:**

At baseline and 6-month follow-up, a total of 153 participants had BMI ≥ 25 and received a completed assessment of behavioral stages. Participants’ mean age was 74.2 years. A reduction of BMI from 31.7 (obese) at baseline to 29.8 (overweight) at 6-months (p=0.0008) was observed. For every stage of improvement in diet, there was a reduction of 1.24 points in BMI (p=0.008). For every stage of progress in exercise, there was a reduction of 0.77 points in BMI (p=0.013).

**Conclusion:**

Pharmacists-led in-home consultations coupled with telephone follow-ups and health education strategies may improve lifestyle and lower BMIs in this cohort. Further studies are needed to investigate these strategies on weight management in geriatric patients with chronic illnesses.

## 1. INTRODUCTION

Obesity in the United States continues to increase and is defined as having a body mass index (BMI) of 30kg/m^2^ or higher [1]. Nationally, African-Americans (AA) have the highest prevalence of obesity (49.6%), compared to Hispanics (44.8%) and non-Hispanic whites (42.2%) [2]. The age-adjusted prevalence of obesity among adults, age 60 and older, in the US is 42.4% [2]. According to the Behavioral Risk Factor Surveillance Survey, at least 20% of adults in each state across America self-report as obese, with 34% of Texans self-report as obese [1]. In Texas, the prevalence of self-reported obesity among African-Americans is 7% higher than non-Hispanic whites (38% vs. 31%, respectively) [1].

Obesity is associated with high risk of development of arterial hypertension. Previous evidence finds that there are short and long-term benefits of weight loss on reducing blood pressure [3,4]. A meta-analysis of randomized controlled trials revealed that each kilogram of weight loss was associated with about a 1 mm Hg reduction in both systolic and diastolic blood pressures [5]. In addition, weight loss can also reduce the risk for cardiovascular disease, stroke, type 2 diabetes, cancer, and cognitive decline [6].

Given that AAs are disproportionately affected by obesity and uncontrolled blood pressure in the US, they have the highest mortality rates of cardiovascular disease (CVD) and stroke, heart failure, and chronic renal disease [7]. Those aged 65 and older experience the highest prevalence of hypertension [8]. The complications of hypertension can be reduced by adopting lifestyle modifications such as healthy eating and physical activity [9].

With the growth in the US aging population, exploring strategies to effectively reduce obesity in this population will contribute to the quality of life of Americans age 65 and older. Specific behavioral models such as the Transtheoretical Model of Change (TTM) have shown promise in improving weight loss [10,11]. The TTM assumes that individuals undergo a series of decisions and actions mediated by specific program strategies or processes that move them from one stage to another [12]. For example, an individual in the pre-contemplation stage has no intention of changing behavior. During contemplation, an individual begins to think about behavior change, but takes no action toward making the change. There is an intention to act within the next 30 days, along with behavioral steps toward the change at the preparation stage. Adopting the new behavior occurs in the the action stage, and the maintenance stage is characterized by continuous action for 6 or more months.

The Managing My Blood Pressure (My BP) program was a pharmacist-led TTM cardiovascular risk reduction program, including hypertension management, medication adherence, and BMI levels [13]. The objective of this study was to investigate the effect of My BP program, home-based pharmacy, and health education intervention on body mass index (BMI) in a cohort of hypertensive overweight African-Americans age 65 and older. We hypothesized that a pharmacist-led home-based intervention that promotes lifestyle modifications would reduce BMI for hypertensive overweight elderly AAs.

## 2. METHODS

### 2.1 Data

The My BP program, funded by the Centers for Medicaid and Medicare Services, was a 6-month prospective, community-based intervention study conducted to investigate the effect of pharmacists’ home visits to AA patients aged 65 years or older with hypertension in Houston, TX over 2 years [13]. Conducted by researchers at Texas Southern University, details of medication management interventions and blood pressure outcomes have been discussed previously [13]. Each participant received an initial pharmacist-led home visit, followed by bi-weekly telephone calls, health education strategies, and a 6-month pharmacist home visit. The baseline and 6 months BMIs were calculated using participants’ self-reported weight (in pounds) and height (in feet and inches) provided to pharmacists at each home visit. Pharmacists and pharmacy interns recorded the stages of behavioral change in diet and exercise into the patient record at each home visit and telephone encounter. The data included in this analysis contained participants’ BMI, an assessment of participants’ stages of behavioral change in diet and exercise, and participant demographic data.

### 2.2 Study Population

Eligibility for the study included self-reporting as AA, age 65 or older, currently receiving Medicare, clinical diagnosis of hypertension, and taking at least one antihypertensive medication, living independently, and having access to a telephone. Three hundred and fifty study participants were recruited through community and faith-based networks throughout Houston over the three-year study period.

### 2.3 Diet and Exercise Interventions

The MyBP project was implemented by an interdisciplinary team of professionals. Licensed pharmacists from ambulatory and clinical settings were hired to counsel participants about diet and exercise during home visits. Each pharmacist received a 3-hour health promotion training highlighting resources available for lifestyle modifications. Pharmacist interns (fourth-year pharmacy students) conducted bi-weekly phone calls. A master-level health educator was hired by the program to provide guidance in health promotion and daily management of the program.

The TTM was used to assess each participants’ readiness to adopt healthy lifestyle changes and provide strategies to guide participants through the stages of change. The My BP program used four of the ten strategies outlined in the TTM model (consciousness-raising, stimulus control, and helping relationship) [12]. Consciousness-raising focuses on increasing awareness of the causes and consequences of risky behavior. Stimulus control removes cues for unhealthy habits and adds prompts for healthier alternatives. Helping relationships combine caring, trust, openness, and acceptance, as well as support for health behavior change [12].

During the first home visit, pharmacists tailored their counseling to each patient’s current clinical needs and progress or regression in their readiness to change. Pharmacists assessed each patient’s readiness to adopt or maintain their level of exercise and healthy eating by asking participants to indicate their readiness to change as pre-contemplation (not intending to change behavior in the next 6 months); contemplation ( strongly inclined to change behavior in the next 6 months); preparation (intend to act in the near future or within next 6months); action (already incorporating behavior for at least 6months); and maintenance (action already happening for over 6 months and the little chance of returning to the old behavior) [12]. The pharmacists reviewed patients’ self-reported BMI, physical activity, and diet and worked with each patient to develop a plan of action. At the end of the home visit, the pharmacist evaluated the patient information and provided recommendations for diet and exercise. Health education materials were used to reinforce pharmacists’ recommendations for healthy eating and exercise.

A series of strategies were used to keep participants engaged, prevent regression, and promote motivation to adopt exercise and healthier eating. Based on the pharmacist’s initial assessment of the participant’s readiness to change, pharmacy interns called each participant bi-weekly. The interns discussed lifestyle modification strategies with participants using a standardized protocol. At each encounter, pharmacy interns encouraged participants to adopt a healthier lifestyle, praised their effort to make a change, and documented the participants’ stage of change. Any medication-related questions were relayed to the registered pharmacists who followed up directly with participants. The health educator maintained a telephone helpline for patients to provide general resources about healthy lifestyle modifications to any participants that called between pharmacists’ home-visits or bi-weekly telephone calls. Two newsletters (See Figures 1.1 and 1.2) and three postcards, including tips on blood pressure monitoring, exercising, reading food labels, and following the DASH diet, were sent to each participant as indicated by pharmacists’ notes in the patient records following home visits and bi-weekly calls. The licensed pharmacists conducted 6-month follow-up visits, documenting participants’ self- reported height and weight for calculation of BMI and staging participants’ readiness to adopt or maintain exercise and/or healthy eating behavior

### 2.4 Statistics

Demographics of study participants were described as averages and percentages. The differences in BMI from baseline to 6 months were analyzed using the student 2-paired t-test. The correlation between stages of changes and BMI was tested using linear regression analysis. A p-value of less than 0.05 was considered statistically significant.

## 3. RESULTS

Demographics of participants are reported in Table 2. A total of 153 older AAs (age 64–96) with hypertension self- identified with a BMI of 25 or above. Each participant received a complete assessment of diet and exercise at baseline and 6-month follow-up home visits. The average age was 74.2 years. More than half had a 12^th^ grade or lower education and were considered low-income (<US$25,000 per year) [14]. The most common co-morbidity was dyslipidemia (50.95%), followed by arthritis (35.9%).

The baseline BMI average was 31.7 (median 31; range 25–58), and the 6-month BMI average was 29.8 (median 30; range 20–58). The difference in BMI at baseline and 6-month follow-up were statistically significant (p=0.0008). Fifty-three out of 153 (34.6%) participants’ BMI was reduced, with an average reduction of 2.4 (median 2, range 1 to 5; n=53).

The number and percentage of participants in each stage of behavioral change are reported in Table 3. There was an overall progression trend from pre-contemplation and contemplation to preparation, action, and maintenance in adherence to pharmacists’ dietary recommendations. At the end of the intervention, the percent of participants in action phase improved from 9.2% at baseline to 20.9% at 6 months (p=0.004). From baseline to 6-month follow-up, there was a 10.2% increase in the percentage of participants in the maintenance phase; however, the results were not statistically significant (p=0.08). Linear regression analysis showed that for every stage of improvement in diet, there was a 1.24 reduction of BMI in 6 months (P=0.008). In other words, if a participant moved from preparation to action, it is estimated that the change resulted in a 1.24 reduction in participants’ BMI.

The differences in the number of participants in each stage of change in exercise were not statistically significant from baseline to 6 months. Linear regression analysis showed for every stage of improvement in exercise, there was a reduction of 0.77 in 6 months BMI. (p=0.013).

## 4. DISCUSSION

African Americans are disproportionally affected by hypertension and obesity. The cause of this problem can be multifaceted, including genetic predispositions, [15] food culture and customs, cost and availability of healthy food,[16] socioeconomic status, and discrimination [17]. This study was a sub-analysis of the My BP program, which found pharmacists’ home visits were associated with improved medication adherence and systolic blood pressure [13]. Our study finding demonstrated that pharmacist home visits, in collaboration with health education strategies based on TTM model, resulted in significant improvement in BMI in participants who were classified as overweight or obese. A literature review indicated that several studies had been conducted to increase exercise and adopt a healthier diet among AA patients; however, few studies focused on low-income, older AA patients. The average age of the study cohort was 74 years. The majority of the study population was low income (69.9%) with annual income < US$24,999 and low education (62.1% completed 12^th^ grader or lower) [15–20].

Although the pharmacist encounter and health education materials emphasized recommendations for exercise, there were no significant differences in stages of change in exercise from baseline to 6- month home visits. Rimando et al. reported that lack of money, lack of motivation to exercise, fear of falls, and injury were barriers to lifestyle modifications in a cohort of older AA patients with hypertension [21]. This population also had a high prevalence of osteoarthritis/ degenerative joint disorder (35.9%), a potential barrier to physical activity coupled with the fear of falling. Lack of physical activity remains a challenge for overweight older AA patients, particularly those with multiple co-morbidities for cardiovascular risks. Future studies are needed to explore factors that motivate this population to engage in physical activities that are safe and prevent injury.

This study demonstrated that the stages of change in diet and exercise using the TTM were significantly correlated with participants’ BMIs. Those who were in the action and maintenance phase were more likely to have a lower BMI at program completion. Past literature reported mixed findings on the usefulness of TTM in predicting weight loss [22,23]. Vallis et al validated the TTM in estimating lifestyle habits in a cohort of 768 patients who were overweight. [22]. Two meta-analyses reported that the TTM stages of change resulted in a minimal difference in weight loss; however, the TTM was significantly associated with improvements in physical activity and dietary change [24,25]. In both studies, the authors reported inconsistent assessment of stages and excessive self-reported measures, including diet and exercise. Weight changes accounted for some of the biases and impressions in using TTM to predict weight loss [24,25].

This study resonates with previous findings that pharmacist home visits, along with the use of health education strategies, resulted in decreases in BMI and improvements in health behavior [26]. Additionally, most previous studies were conducted in groups, while this study offered individualized approaches (i.e, in-home and telephone counseling and newsletters) and recommendations to a cohort of older AA patients, most with multiple chronic co-morbidities (e.g. hypertension, falls, hypoglycemia, arthritis, dementia, and depression).

Health professionals such as pharmacists, who understand the value of the TTM, can be effective in helping patients lose weight. During the past century, the role of pharmacists has evolved from dispensing medications in retail and hospital pharmacies to providing lifestyle modification counseling in community settings, including home settings [13,25]. Pharmacists’ position as the most frequented health providers in communities also make them easily accessible and keenly aware of patients’ needs [26].

There are several limitations of this study. One limitation is that weight and height data used to calculate the BMI and stages of change was based on self-reported history provided by the patients at baseline and 6 months. It was possible that participants reported this data in error. Additionally, there was no randomization or control group. This study was a pre- and post-intervention analysis using a convenience sample, restricting generalization of results to similar or other cohorts. Further studies are needed to investigate the impact of using an enhanced controlled care group to determine the true difference. It should also be noted that there were several confounders that were not included in the analysis that may impact the proposed effectiveness of the study. Lastly, at only six months duration, participants’ movement through the stages was limited from preparation to action in diet. Perhaps, a 2-year intervention period would capture the long-term effect of the pharmacist home visits and health educator interventions.

## 5. CONCLUSION

Despite these limitations, this study demonstrated that pharmacist home visits, followed by telephone calls and health educator support, resulted in dietary improvements and lowered BMI in a cohort of overweight AA older patients with hypertension. Further studies are needed to examine the combined role of pharmacists and health educators in motivating patients in this population to progress through the stages of change.

## Figures and Tables

**Table 1.** TTM Process and My BP Program Strategies

TTM Processes of Change	Process of Change Strategies Used in My BP Program
**Consciousness-raising** focuses on increasing awareness of the causes and consequences of risky behavior.	Home visits, bi-weekly phone calls, and newsletters focused on the causes and consequences of hypertension, emphasizing its relationship to BMI and unhealthy eating, and lack of exercise.
**Stimulus control** removes cues for unhealthy habits and adds prompts for healthier alternatives.	Pharmacists reviewed the DASH diet with patients. Newsletters included AA-inspired cooking recipes. In addition, postcards containing DASH diet resources and reminders to eat healthy were mailed out to patients.
**Helping relationships** combine caring, trust, openness, and acceptance, as well as support for health behavior change.	Pharmacists, pharmacy interns, and the project’s health educator were instrumental in building rapport with the patients. Fostered trust by hiring pharmacists with proven experience working with AA seniors. Health educator trained pharmacy interns to effectively communicate lifestyle modification information to the patients. Health educators’ help line addressed additional concerns and questions.

**Table 2.** Patient Demographics

Demographics	N=153

Age, mean (range)	74.2 (64–96)

Sex, no. (%)	125 (81.7%)

Smoking history, no. (%)	26 (17%)

Regular alcohol use, no. (%)	44 (28.8%)

Illicit drug use, no (%)	2 (1.3%)

Years of hypertension diagnosis, mean (range)	17.9 (1–60)

Number of antihypertensive medication use, mean (range)	2.1 (1–5)

Co-morbid conditions	
- Arthritis/ Degenerative joint diseases	55 (35.9%)
- Diabetes	44 (28.8%)
- Dyslipidemia	78 (50.9%)

Education	
• 12^th^ grade or lower	95 (62.1%)
• Associate degree/ Certification	25 (16.3%)
• 4 year college degree	15 (9.8%)
• Graduate school	10 (6.5%)

Income	
• US $0 to $24,999	107 (69.9%)
• US $25,000 to $49,999	25 (16.3)
• US $50,000 to $74,999	7 (4.6%)
• US $75,000 or more	3 (2.0%)

**Table 3.** My BP Participants’ Stages of Change at Baseline and 6 month Follow-up

	Pre-contemplation	Contemplation	Preparation	Action	Maintenance
**Diet**					
Baseline, n(%)	10 (6.5%)	11 (7.2%)	33 (21.6%)	14 (9.2%)	83 (54.2%)
6 months, n(%)	3 (2.0%)	7 (4.6%)	9 (5.9%)*	32 (20.9%)**	98 (64.0%)
**Exercise**					
Baseline, n(%)	11 (7.2%)	12 (7.8%)	28 (18.3%)	15 (9.8%)	80 (52.3%)
6 months, n(%)	15 (9.8%)	14 (9.2%)	19 (12.4%)	11 (7.2%)	88 (57.5%)

* p=0.0001

** p=0.04

*** p=0.01

## References

[1] Centers for Disease Control. Adult Self-Reported Obesity Maps. Available:https://www.cdc.gov/obesity/data/prevalence-maps.html. 2021(9). Accessed 10 June 2021.

[2] Hales CM, Carroll MD, Fryar CD, Ogden CL. Prevalence of Obesity and Severe Obesity Among Adults: United States. 2017–2018. NCHS Data Brief. No. 360. 2, 2020. Available: https://www.cdc.gov/nchs/data/databriefs/db360-h.pdf. Accessed 10 June 2021. 32487284

[3] Elmer PJ, Obarzanek E, Vollmer WM, Effects of comprehensive lifestyle modification on diet, weight, physical fitness, and blood pressure control: 18-month results of a randomized trial. Ann Intern Med. 2006;144:485–495. 16585662 10.7326/0003-4819-144-7-200604040-00007

[4] He J, Whelton PK, Appel LJ, Charleston J, Klag MJ. Long-term effects of weight loss and dietary sodium reduction on incidence of hypertension. Hypertension. 2000;35:544–549. 10679495 10.1161/01.hyp.35.2.544

[5] Neter JE, Stam BE, Kok FJ, Influence of weight reduction on blood pressure. Hypertension. 2003;42:878–884. 12975389 10.1161/01.HYP.0000094221.86888.AE

[6] DeckersK, Van BoxtelMPJ, VerheyFRJ, Obesity and cognitive decline in adults: effect of methodological choices and confounding by age in a longitudinal study. J Nutr Health Aging. 2017;21(5):546–553.2844808510.1007/s12603-016-0757-3

[7] Stewart J, Manmathan G, & Wilkinson P. Primary prevention of cardiovascular disease: A review of contemporary guidance and literature. JRSM Cardiovascular Disease. 2017;6:1–9. 2048004016687211. Avaialble:10.1177/2048004016687211 PMC5331469 28286646

[8] Merai R, Siegel C, Rakotz M, CDC Grand Rounds: A Public Health Approach to Detect and Control Hypertension. MMWR Morb Mortal Wkly Rep. 2016;65(45):1261–1264. 27855138 10.15585/mmwr.mm6545a3

[9] Centers for Disease Control and Prevention. (2020, 2 24). Prevent high blood pressure. Centers for Disease Control and Prevention. Avaialble:https://www.cdc.gov/bloodpressure/prevent.htm. Accessed 21 September 2021.

[10] Johnson SS, Paiva AL, Cummins CO, Johnson JL, Dyment SJ, Wright JA, Prochaska JO, Prochaska JM, Sherman K. Transtheoretical Model-Based Multiple Behavior Intervention for Weight Management: Effectiveness on a Population Basis. Prev Med. 2008;46(3): 238–246. DOI: 10.1016/j.ypmed.2007.09.010 18055007 PMC2327253

[11] de Freitas PP, de Menezes MC, dos Santos LC, The transtheoretical model is an effective weight management intervention: a randomized controlled trial. BMC Public Health. 2020;20:652. Avaialble:10.1186/s12889-020-08796-1 32393214 PMC7216547

[12] Prochaska JO, Redding CA, Evers KE. The Transtheoretical Model and Stages of Change. Health Behavior and Health Education, Theory, Research and Practice. 4^th^ Edition. Jossey-Bass Publishing. 2008;135–160.

[13] Moultry AM, Pounds K, Poon I. Managing medication adherence in elderly hypertensive patients through pharmacist home visits. Consultant Pharmacist. 2015;30(12):710–9. DOI: 10.4140/TCP.n.2015.710 26671271

[14] Congressioal Budget Office. The Distribution of Household Income and Federal Taxes; 2013. 2016(June). Avaialble:https://www.cbo.gov/sites/default/files/114th-congress-2015-2016/reports/51361-householdincomefedtaxesonecol.pdf. Accessed 15 October 2021.

[15] Bertoni AG, Foy CG, Hunter JC, A multilevel assessment of barriers to adoption of DASH among African Americans of low socioeconomic status. J Health Care Poor Underserved. 2011; 22(4):1205–20. 22080704 10.1353/hpu.2011.0142 PMC3769217

[16] Forsyth JM, Schoenthaler A, Ogedegbe G, Ravenell J. Perceived racial discrimination and adoption of health behaviors in hypertensive BlackAmericans: the CAATCH trial. J Health Care Poor Underserved. 2014;25(1):276–91. 24509026 10.1353/hpu.2014.0053 PMC12516789

[17] Blanks SH, Treadwell H, Bazzell A, Community Engaged Lifestyle Modification Research: Engaging Diabetic and Prediabetic African American Women in Community-Based Interventions. J Obes. 2016;3609289. 27493797 10.1155/2016/3609289 PMC4963564

[18] Kegler MC, Haardorfer R, Alcantara IC, Gazmararian JA, Veluswamy JK, Hodge TL, Impact of Improving Home Environments on Energy Intake and Physical Activity: A Randomized Controlled Trial. Am J Public Health. 2016; 106(1):143–52. 26696290 10.2105/AJPH.2015.302942 PMC4695922

[19] Rimando M Perceived Barriers to and Facilitators of Hypertension Management among Underserved African American Older Adults. Ethn Dis. 2015;25(3):329–36. 26675535 10.18865/ed.25.3.329 PMC4671409

[20] Sattin RW, Williams LB, Dias J, Williams LB, Dias J, Garvin JT Community Trial of a Faith-Based Lifestyle Intervention to Prevent Diabetes Among African-Americans. J Community Health. 2016; 41(1):87–96. 26215167 10.1007/s10900-015-0071-8 PMC4715566

[21] Vallis M, Ruggiero L, Greene G, Jone H, Zinman B, Rossi S, Stages of change for healthy eating in diabetes: relation to demographic, eating-related, healthcare utilization, and psychosocial factors. Diabetes Care. 2003;26(5):1468–74. 12716806 10.2337/diacare.26.5.1468

[22] Mastellos N, Gunn LH, Felix LM, Car J, Majeed A, Transtheoretical model stages of change for dietary and physical exercise modification in weight loss management for overweight and obese adults. Cochrane Database Syst Rev. 2014;(2):CD008066. 24500864 10.1002/14651858.CD008066.pub3 PMC10088065

[23] Tuah NA, Amiel C, Qureshi S, Car J, Balvinder K, Majeed A, Transtheoretical model for dietary and physical exercise modification in weight loss management for overweight and obese adults. Cochrane Database Syst Rev. 2011;(10):CD008066. 21975777 10.1002/14651858.CD008066.pub2

[24] Jordan M, Harmon J. Pharmacists intervention for obesity: improving adherence to patient outcomes. Integrated Pharmacy Research and Practice. 2015;5:79–89. 10.2147/IPRP.S72206 PMC5741031 29354522

[25] Bennett M, Goode JV J Am Pharm Assoc (2003).-Recognition of community-based pharmacist practitioners: Essential health care providers. 2016; 56(5):580–3. 27594108 10.1016/j.japh.2016.04.566

[26] Moose J, Branham A. Pharmacists as Influencers of Patient Adherence. Pharmacy Times; 2014. Avaialble:https://www.pharmacytimes.com/publications/directions-in-pharmacy/2014/august2014/pharmacists-as-influencers-of-patient-adherence-. Accessed 13 April 2019.

[27] Nadkarni GN, Galarneau G, Ellis SB, Apolipoprotein L1 variants and blood pressure traits in African Americans. J Am Coll Cardiol. 2017; 69(12):1564–1574. 28335839 10.1016/j.jacc.2017.01.040 PMC5479565

